# Supplementary material for: The interactome of intact mitochondria by cross-linking mass spectrometry provides evidence for coexisting respiratory supercomplexes
Source: Mol Cell Proteomics. 2017 Dec 8;17(2):216–32. doi: 10.1074/mcp.RA117.000470 (PMC5795388; doi:10.1074/mcp.RA117.000470)
Supplement: Supplemental Data [file supp_RA117.000470_133922_0_supp_23978_fzffwf.zip › spectra_annotation/mito_DR_spectra_annotation/458-1-13-1-13-1.pdf]

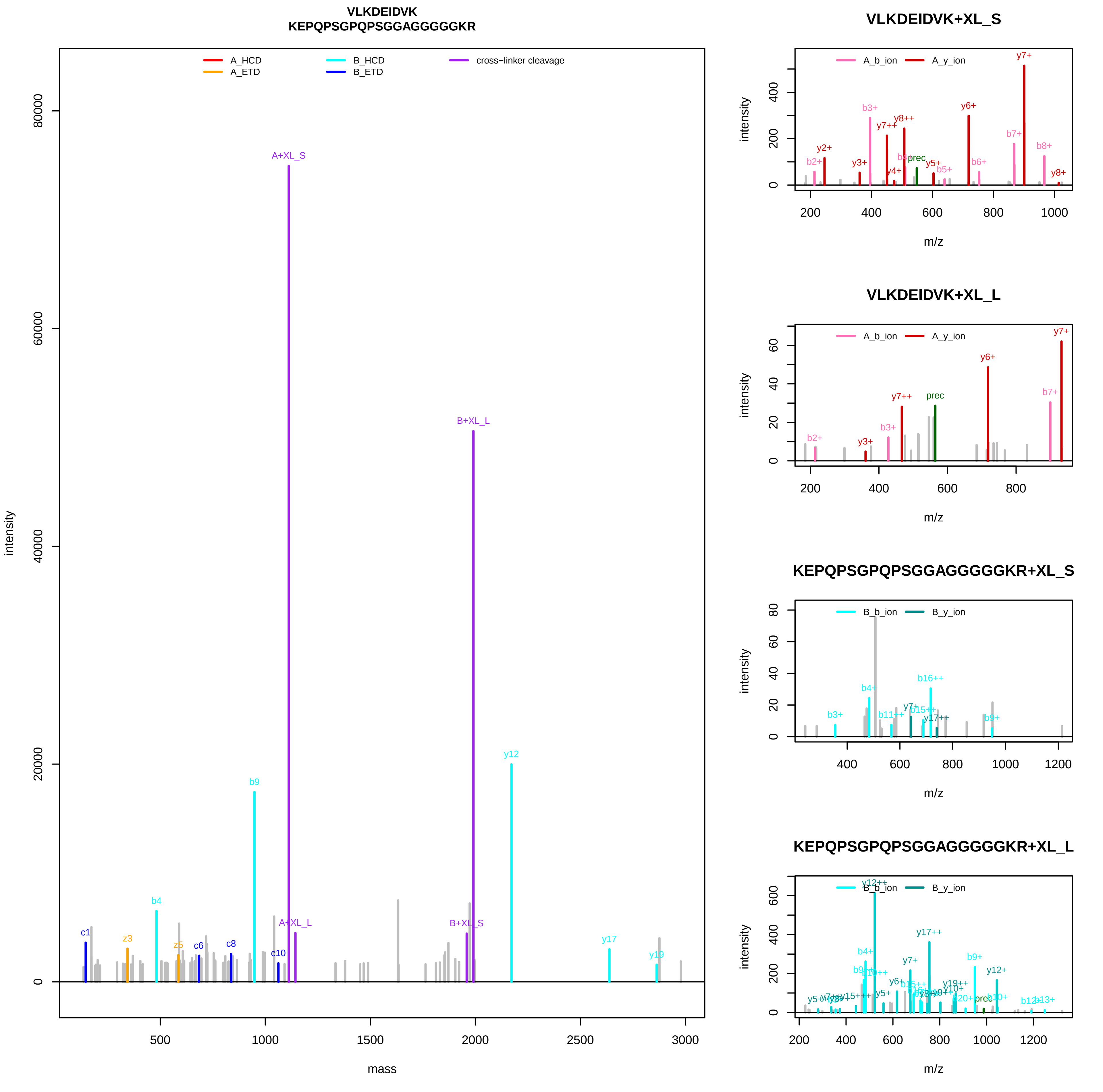
VLKDEIDVK
KEPQPSGPQPSGGAGGGGGKR
A_HCD
A_ETD
B_HCD
B_ETD
cross−linker cleavage
intensity
mass
A+XL_S
B+XL_L
A+XL_L
B+XL_S
b4
b9
y12
y17
y19
c1
c6
c8
c10
z3
z5
VLKDEIDVK+XL_S
VLKDEIDVK+XL_L
KEPQPSGPQPSGGAGGGGGKR+XL_S
KEPQPSGPQPSGGAGGGGGKR+XL_L
A_b_ion
A_y_ion
B_b_ion
B_y_ion
m/z
prec
b2+
b3+
b6+
b7+
b8+
y2+
y3+
y4+
y5+
y6+
y7+
y7++
y8++
y8+
b4+
b11++
b15++
b16++
b9+
y17++
y12++
y19++
y12+
